# The Effect of Walking and Teleportation on Spatial Updating in Virtual and Real Scenes

**DOI:** 10.1068/ig7

**Published:** 2013-10-01

**Authors:** J Vuong, L C Pickup, A Glennerster

**Affiliations:** University of Reading, UK; University of Reading, UK; University of Reading, UK

## Abstract

Intuitively, it seems as if we should be able to point accurately to the location of a target object within a room even if we were teleported to a different location and the object removed from view. We measured the precision of pointing to a previously-seen object in a real room, a virtual room with same dimensions (presented in immersive virtual reality) and a sparse virtual scene consisting only of long thin poles at the same locations as the target object and room corners. Participants viewed the target object from one location, walked to another so that the object passed out of view, then turned in complete darkness to point at the location of the previously-viewed target. In a separate experiment, participants viewed a sparse scene consisting of long thin poles (including a target) and had to point to the location of the absent target after teleportation to a new location within the scene. Pointing precision in this case was dramatically reduced (σ ≈ 34°) compared to the conditions in which participants walked in the real room, virtual room or sparse scene. In the latter three conditions, pointing precision was very similar (σ ≈ 15°) despite the removal of prominent distance cues in the sparse condition. Our results show that spatial updating after teleportation is substantially poorer than when walking between two locations. [Supported by Microsoft Research and Wellcome Trust]

